# Ceftazidime-Avibactam Versus Colistin for the Treatment of Multidrug-Resistant *Pseudomonas aeruginosa* Infections: A Multicenter Cohort Study

**DOI:** 10.3390/ph18010108

**Published:** 2025-01-16

**Authors:** Thamer A. Almangour, Zakiyah Alkherb, Leen Ghonem, Mohammed Al Musawa, Abdullah Almohaizeie, Sara Almuhisen, Aminah Alharbi, Nader Damfu, Doaa Aljefri, Jeelan Alghaith, Awaly Alfozan, Ahlam Alghamdi, Ahmad Aljabri, Abdullah A. Alhifany, Mohammed Alessa, Yazed Saleh Alsowaida

**Affiliations:** 1 Department of Clinical Pharmacy, College of Pharmacy, King Saud University, P.O. Box 2457, Riyadh 11451, Saudi Arabia; 2 Clinical Pharmacy Services, King Saud University Medical City, King Saud University, Riyadh 11451, Saudi Arabia; 3 Pharmaceutical Care Division, King Faisal Specialist Hospital & Research Centre, Jeddah 23433, Saudi Arabiaaminah.m.alharbi@gmail.com (A.A.); daljefri@kfshrc.edu.sa (D.A.); 4 Anti-Infective Research Laboratory, Department of Pharmacy Practice, Eugene Applebaum College of Pharmacy and Health Sciences, Wayne State University, Detroit, MI 48201, USA; 5 Pharmaceutical Care Division, King Faisal Specialist Hospital & Research Centre, Riyadh 12713, Saudi Arabia; amohaizeie@kfshrc.edu.sa (A.A.);; 6 College of Pharmacy, Alfaisal University, Riyadh 11533, Saudi Arabia; 7 Pharmacy Services Administration, King Fahad Medical City, Riyadh 12231, Saudi Arabia; 8 King Abdullah International Medical Research Centre, Riyadh 11481, Saudi Arabia; damfona@ngha.med.sa; 9 Infection Prevention and Control Department, King Abdulaziz Medical City, Ministry of National Guard Health Affairs, Jeddah 11426, Saudi Arabia; 10 King Saud bin Abdulaziz University for Health Sciences, Jeddah 22384, Saudi Arabia; 11 Department of Pharmacy Practice, College of Pharmacy, Princess Nourah bint Abdulrahman University, P.O. Box 84428, Riyadh 11671, Saudi Arabia; 12 Pharmaceutical Care Department, King Salman bin Abdulaziz Medical City, Madinah 42319, Saudi Arabia; 13 Pharmacy Practices Department, College of Pharmacy, Umm Al-Qura University, Makkah 24382, Saudi Arabia; 14 Department of Clinical Pharmacy, College of Pharmacy, University of Ha’il, P.O. Box 6166, Hail 81442, Saudi Arabia

**Keywords:** *P. aeruginosa*, multidrug-resistant, ceftazidime-avibactam, colistin

## Abstract

**Purpose:** To evaluate the real-world evidence of ceftazidime-avibactam (CAZ-AVI) compared to intravenous colistin for the treatment of multidrug-resistant (MDR) *P. aeruginosa* infections. **Method:** This is a multicenter, retrospective cohort study conducted in the period between 2017 and 2023 at five institutions for patients who received either CAZ-AVI or colistin-based regimens for treating MDR *P. aeruginosa* infections. Outcomes were compared using multivariate logistic regression analysis. **Result:** Among the screened patients, 203 patients were included: 89 in the CAZ-AVI group and 114 in the colistin group. A total of 57% presented with pneumonia, 21% with bacteremia, and 61% were in the intensive care unit. The rate of clinical cure was significantly higher among patients who received CAZ-AVI (67% vs. 50%; OR, 2.07; 95% CI, 1.16–3.68). The rate of in-hospital mortality was numerically lower among patients who received CAZ-AVI (40% vs. 49%; OR, 0.58; 95% CI, 0.33–1.03). The rate of AKI was significantly lower among patients who received CAZ-AVI (15% vs. 43%; OR, 0.23; 95% CI, 0.11–0.45). **Conclusion:** CAZ-AVI was more effective in treating MDR *P. aeruginosa* infections and showed a better safety profile compared to colistin. Thus, CAZ-AVI could be a better alternative for treating MDR *P. aeruginosa* infections.

## 1. Introduction

With advancements in antimicrobial resistance, resistant Gram-negative pathogens add complexity to patient care. *Pseudomonas aeruginosa* is widely recognized as a challenging healthcare-associated Gram-negative pathogen given its ability to develop resistance to the most routinely used antibiotics [1]. In 2024, carbapenem-resistant *P. aeruginosa* was designated as a high priority for development and research by the World Health Organization [2]. The global rate of multidrug-resistant (MDR) *P. aeruginosa* is around 25% according to the SENTRY Antimicrobial Surveillance Program [3]. In Saudi Arabia, antibiotic-resistant *P. aeruginosa* is also a concern [4,5]. Data from published literature, as well as the Global Antimicrobial Resistance Surveillance System, demonstrated that the rate of carbapenem-resistant *P. aeruginosa* in Saudi Arabia is reaching up to 30% [6,7].

Options to treat MDR *P. aeruginosa* infections are limited, which include colistin as a treatment of last resort. However, the use of intravenous (IV) colistin is discouraged due to its mediocre pharmacokinetics profile [8,9], risk of acute kidney injury [10,11], complex dosing regimens, and issues with reliable in vitro susceptibility testing [12,13]. Therefore, there is a need for novel agents to treat infections caused by MDR *P. aeruginosa.*

Avibactam expands the activity of ceftazidime mainly through inhibition of AmpC, a clinically important cephalosporinase, but other resistance mechanisms of *P. aeruginosa* are unlikely to be impacted. Although this may explain the lower potency of ceftazidime-avibactam (CAZ-AVI) versus the other novel agent ceftolozane-tazobactam (C-T), CAZ-AVI is still active against MDR strains of *P. aeruginosa* and serves as a potential option, especially during the shortages or a global recall of the latter agent [14]. Notably, the majority of the hospitals in Saudi Arabia have CAZ-AVI as a formulary drug as opposed to C-T.

Clinical trials, which led to the US Food and Drug Administration approval of CAZ-AVI, included only a small number of infections caused by *P. aeruginosa*. Further, the MDR isolates in these trials were limited [15]. Therefore, these trials did not address patients most in need of CAZ-AVI. In addition, pooled data from five Phase III clinical trials that evaluated the clinical activity of CAZ-AVI versus more traditional regimens against MDR pathogens including *P. aeruginosa* are available [16]. However, colistin was part of only one out of these five trials; *P. aeruginosa* represented <10% of the total number of isolates, and only 66% were susceptible to CAZ-AVI. Lastly, unlike the available data for C-T [17,18], no study was designed to investigate the clinical activity of CAZ-AVI versus more traditional antibiotics to treat infections caused by MDR *P. aeruginosa*. To fill this gap, we conducted this study to compare CAZ-AVI and IV colistin to treat infections caused by MDR *P. aeruginosa.*

## 2. Results

Overall, 203 patients met our inclusion criteria: 89 in the CAZ-AVI group and 114 in the colistin group. The mean age was 60 ± 18 years and 120 (59%) patients were male. More than half of the study population was diabetic (*n* = 123; 61%). Other common comorbidities included hypertension (*n* = 116; 57%) and immunosuppression (*n* = 57; 28%). The median (IQR) CCI for all eligible patients was 5 (2–7). One hundred and twenty-three (61%) patients were in ICU settings, and 84 (41%) were mechanically ventilated. Hospital-acquired pneumonia and ventilator-associated pneumonia were the most common source of infection (*n* = 115; 57%). Bacteremia was documented in 43 (21%) patients. Polymicrobial infection occurred in 97 (48%) patients. Most baseline characteristics were balanced between the 2 groups (Table 1).

For CAZ-AVI, the median (IQR) time to active therapy and to the study drug were 85 (16–120) hours and 100 (30–155) hours, respectively. In contrast, for colistin, the median (IQR) times to active therapy and to the study drug were 72 (10–144) hours and 72 (24–156) hours, respectively. Of note, differences were not statistically different. More patients in the colistin group received combination therapy (86% versus 19%; *p* < 0.001). In the CAZ-AVI group, specific MIC data were available in 44 cases and the median MIC was 4 µg/mL (range 2 to 8 µg/mL). The numbers of isolates with CAZ-AVI MICs of 2, 4, 6, and 8 µg/mL were 17, 14, 1, and 12, respectively.

In-hospital mortality (40% vs. 49%; *p* = 0.060; OR, 0.58; 95% CI, 0.33–1.03) was numerically lower for CAZ-AVI recipients than colistin recipients. Clinical cure (67% vs. 50%; *p* = 0.013; OR, 2.07; 95% CI, 1.16–3.68) was significantly more common in patients who received CAZ-AVI even after adjusting for differences between the two groups. The rate of AKI (15% vs. 43%; *p* < 0.001; OR, 0.23; 95% CI, 0.11–0.45) was significantly lower in CAZ-AVI group even after adjusting for differences between the two groups. Regarding other outcomes, differences between the two groups were not statistically different, including microbiologic eradication, infection-related mortality, 30-day readmission, 30-day recurrence, 90-day recurrence, length of hospital and ICU stay from the onset of infection, and the duration of mechanical ventilation (Table 2).

### Subgroup Analysis

**Figure 1:**
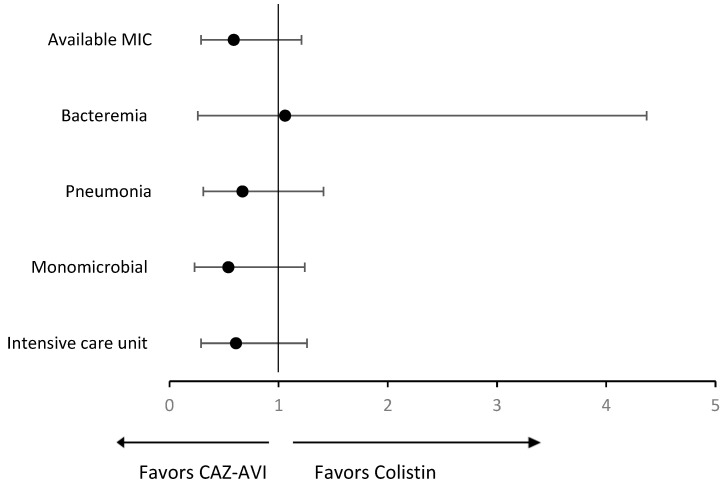
Odds ratios of overall in-hospital mortality for CAZ-AVI versus colistin among subpopulations of interest. Forest plot of boxes depicting odds ratios with 95% confidence intervals shown as horizontal lines.

Among cases with available MIC for CAZ-AVI, the difference was statistically significant between those who received CAZ-AVI versus colistin in clinical cure (68% vs. 50%; *p* = 0.039). However, no statistically significant differences were observed in the in-hospital mortality (36% vs. 49%; *p* = 0.149) and infection-related mortality (16% vs. 25%; *p* = 0.240). In the subgroup analysis of bacteremia, the clinical cure was significantly higher in CAZ-AVI compared to colistin (100% vs. 48.5%; *p* = 0.004). However, no statistically significant differences were observed in the in-hospital mortality (50% vs. 48.5%; *p* = 0.933) and infection-related mortality (10% vs. 21.2%; *p* = 0.425). In the subgroup analysis of patients with pneumonia, there were no statistically significant differences in clinical cure (56.5% vs. 44.9%; *p* = 0.223), in-hospital mortality (43.5% vs. 53.6%; *p* = 0.286), and infection-related mortality (19.6% vs. 26.1%; *p* = 0.419) between those who received CAZ-AVI compared to colistin. Furthermore, with monomicrobial infection, the clinical cure was significantly higher in the CAZ-AVI group vs. colistin (76.6% vs. 55.9%; *p* = 0.027). However, no statistically significant differences were observed in the in-hospital mortality (25.5% vs. 39%; *p* = 0.143) and infection-related mortality (12.8% vs. 16.9%; *p* = 0.550). Lastly, when solely including patients in the ICU, no statistically significant differences were observed between those who received CAZ-AVI versus colistin in clinical cure (60% vs. 42.5%; *p* = 0.056), in-hospital mortality (48% vs. 60.3%; *p* = 0.179), and infection-related mortality (18% vs. 28.8%; *p* = 0.172). Odds ratios of overall in-hospital mortality for CAZ-AVI versus colistin among subpopulations of interest are presented in Figure 1.

## 3. Discussion

In this observational study, we compared CAZ-AVI to colistin for the treatment of MDR *P. aeruginosa* infections. Our results showed that CAZ-AVI was associated with a better clinical cure, with a number needed to treat of six. CAZ-AVI was also associated with lower AKI, with a number needed to harm of four. No statistically significant difference was found in other outcomes, including in-hospital mortality, infection-related mortality, microbiologic eradication, 30-day readmission, 30- and 90-day recurrence, length of stay, or the duration of mechanical ventilation. Given the mortality rates of 40% and 49% with OR of 0.58, assuming Alpha of 0.05 and Beta of 0.2, the needed sample size that might detect the difference in the mortality rate is 475 patients in each group.

Although data comparing CAZ-AVI vs. more traditional regimens for the treatment of carbapenem-resistant Enterobacterales are currently available [19,20,21,22,23], data investigating the clinical outcome of CAZ-AVI versus more traditional regimens for the treatment of MDR *P. aeruginosa* are limited, and to our knowledge, no study has yet been designed to address this question. Findings are limited to data pooled from five RCTs, which showed that favorable clinical response was observed in 57% versus 54% in CAZ-AVI versus carbapenem-based comparators, respectively [16]. It should be noted, however, that colistin was part of only one out of five RCTs, *P. aeruginosa* represented <10% of the study population (56 and 39 in CAZ-AVI and comparator groups, respectively), and one-third of the isolates was not susceptible to CAZ-AVI. Our findings are consistent with the studies comparing the other novel antipseudomonal antibiotics, C-T, to more traditional regimens, which showed that C-T was associated with improved clinical cure and decreased AKI [17,18]. CAZ-AVI was also shown to be as effective as C-T for the treatment of *P. aeruginosa* infections, which supports the preferential use of the novel β-lactam β-lactamase inhibitor combination over more traditional regimens [24]. Although not achieving statistical significance, primarily due to the small sample size, the numerical differences suggest that these novel agents decreased the rate of mortality.

The term “difficult-to-treat” resistance (DTR) was introduced earlier [25], and is currently used in the latest Infectious Diseases Society of America guidance [26]. Although our inclusion criterion was based on the presence of MDR strains, these strains can be considered *P. aeruginosa* with DTR, given that the internal protocols in the contributing centers restrict the use of the study drugs to *P. aeruginosa* that is not susceptible to all traditional antipseudomonal β-lactams and antipseudomonal fluoroquinolones (the definition of DTR *P. aeruginosa*) [26].

This study has several limitations including the retrospective observational nature of the design. In addition, as consistent with real-life practice, the MIC data were not reported by all institutions. Moreover, dose-dependent sensitivity analysis was not conducted. Additionally, the sample size was relatively small due to the late approval of CAZ-AVI in Saudi Arabia, the preference of the other novel antibiotics, C-T if available, over CAZ-AVI in some hospitals to treat these infections, as well as the relatively small number of MDR *P. aeruginosa* infections compared to other pathogens. However, to our knowledge, this is the first real-world observational study designed to evaluate the effectiveness and safety of CAZ-AVI vs. colistin for treating MDR *P. aeruginosa* infections. Broth microdilution was used to assess the susceptibility of colistin, which is more reliable compared to the gradient diffusion method. Lastly, the control arm included only one comparator with no major variability in dosing.

In conclusion, for infection caused by MDR *P. aeruginosa*, CAZ-AVI demonstrated preferred outcomes in clinical cure and had lower rate of AKI versus colistin. Thus, CAZ-AVI could be a better alternative for treating MDR *P. aeruginosa* infections.

## 4. Materials and Methods

### 4.1. Patients and Setting

This was a multicenter, retrospective cohort study of patients who received either CAZ-AVI or IV colistin for the treatment of infections caused by MDR *P. aeruginosa* in the period between May 2017 and February 2023. This study was conducted at 5 tertiary care hospitals in Saudi Arabia, 3 in Riyadh city with bed capacity ranging between 1200 and 1600 beds, King Saud University Medical City, King Fahad Medical City, and King Faisal Specialist Hospital and Research Center; 2 hospitals were in Jeddah city with bed capacity ranging between 380 and 750 beds, King Abdulaziz Medical City and King Faisal Specialist Hospital and Research Center. Institutional Review Boards of the participating centers approved this study. We included patients aged ≥18 years, admitted to one of the participating hospitals between 2017 and 2023, who developed an infection due to MDR *P. aeruginosa*, and were treated with either CAZ-AVI or IV colistin for at least 48 h. We excluded patients who received CAZ-AVI and IV colistin concurrently for more than 48 h, or if the isolate of *P. aeruginosa* was confirmed as not susceptible to the drug being evaluated. Only the first episode of MDR *P. aeruginosa* per patient was included. Data were retrieved from electronic health records. Main study outcomes included overall in-hospital mortality, clinical cure at the end of treatment, and acute kidney injury (AKI). Other outcomes included infection-related mortality, microbiologic eradication, 30-day readmission, 30- and 90-day recurrence, length of hospital and intensive-care unit (ICU) stay from the onset of the infection, and duration of mechanical ventilation. The total daily dose of IV colistin was 9 million international units (MIUs) given as a loading dose followed by at least 9 MIUs given and adjusted per renal function. CAZ-AVI was administered intravenously at a dose of 2.5 g every 8 h and adjusted per renal function.

### 4.2. Data Collection

For each eligible patient, the following data were recorded from electronic medical records: demographics, clinical characteristics, laboratory tests at baseline and throughout the treatment course, comorbid conditions including Charlson comorbidity index (CCI), site of infection, time to appropriate antibiotic (any antibiotic with in vitro susceptibility), time to study drug (CAZ-AVI or IV colistin), dosing of CAZ-AVI and colistin, duration of treatment, length of hospital stay, susceptibility to study antimicrobials, combined antibiotics and the susceptibility data, presence of polymicrobial infections, the severity of infection, admission setting, Acute Physiology and Chronic Health Evaluation (APACHE II) score for patients admitted to the ICU, immune status, placement of indwelling devices at the onset of infection, and infectious diseases consultation. Clinical effectiveness, microbiological, and safety outcomes were recorded and assessed.

### 4.3. Microbiological Testing

Microbiologic testing was performed according to each institution’s own protocols. The following automated systems were used based on the institution which included MicroScan WalkAway 96 plus (Beckman Coulter, Inc., Brea, CA, USA), BD Phoenix M50 (Becton Dickinson Diagnostic Systems, Sparks, MD, USA), or the VITEK 2 system (bioMérieux, Marcy-l’Étoile, France). The susceptibility testing of the organisms to colistin was conducted using commercial broth microdilution (ComASP™ Colistin (Liofilchem^®^ srl, Roseto degli Abruzzi, Italy)). Colistin breakpoints and susceptibility interpretive criteria for *P. aeruginosa* were according to the Clinical and Laboratory Standard Institute (CLSI) [27]. Most of the collected data were from the period before applying the most recent CLSI standards, in which the susceptible category for colistin was removed [12]. CAZ-AVI breakpoints and susceptibility interpretive criteria for *P. aeruginosa* were also based on the CLSI as follows: ≤8/4 was susceptible and ≥16/4 was resistant [27]. Susceptibility testing for CAZ-AVI was conducted using gradient diffusion by either Etest (bioMérieux, Marcyl’Étoile, France) or MIC test strip (Liofilchem^®^, Roseto degli Abruzzi, Italy).

### 4.4. Definitions

Sepsis was defined as suspected or documented infection plus an increased Sequential Organ Failure Assessment (SOFA) score of ≥2 from baseline [28]. Septic shock was defined as sepsis with organ dysfunction and consistent hypotension despite proper volume resuscitation, which requires vasopressors and a serum lactate level >2 mmol/L [28]. MDR *P. aeruginosa* was defined as *P. aeruginosa* that was not susceptible to at least 1 agent in ≥3 antimicrobial categories [29]. Time to active therapy was defined as the time from the cultures collected to the time any in vitro active antipseudomonal agent was started. Time to study drug was defined as the time from the cultures collected to the time the study drug was initiated (CAZ-AVI or colistin).

In-hospital mortality was defined as death due to any cause during the same hospitalization. It was considered infection-related if patients had ongoing unequivocal clinical and/or biochemical signs of infection at the time of their death.

Clinical cure was defined as the resolution of symptoms and signs of infection with the study drug without treatment needing to have been modified due to toxicity or failure. We included several factors in the assessment of clinical cure, including normalization of vital signs, white blood cells, C-reactive protein, or procalcitonin, if applicable. Assessment of clinical cure was performed by infectious diseases specialists using a dichotomous variable with “yes” or “no”.

Microbiologic eradication was defined as negative repeated cultures, while persistence was defined as persistent growth of the etiologic pathogen at the same infection site (assessed only if patients had repeated cultures).

Recurrence (30- or 90-day) of infection was defined as a new infection event at the same site due to the same pathogen of the index culture after evidence of at least one negative growth of microorganisms during 30 or 90 days of the primary infection episode.

Polymicrobial infection was defined as the isolation of additional pathogens from the same culture or during the same episode of infection.

AKI was assessed using “RIF” components of RIFLE (Risk, Injury, Failure, Loss of kidney function, and End-stage kidney disease) criteria [30]. We also included whether patients received renal replacement therapy due to AKI.

### 4.5. Statistical Analysis

Descriptive statistics were used to summarize the data. Categorical variables were expressed as numbers and percentages and compared using the χ^2^ test. Continuous variables were presented as mean ± standard deviation (SD) or median and interquartile range (IQR) and compared using an independent t-test or Wilcoxon rank-sum test, as appropriate based on their distribution. Analyses were performed with the level of significance set at *p* < 0.05. The number needed to treat for clinical cure and the number needed to harm for AKI were calculated. Multivariate analysis using logistic regression was used to determine the independent impact of treatment on the outcomes of interest (overall in-hospital mortality, infection-related mortality, clinical cure, and AKI). Along with the treatment groups, relevant demographics and baseline clinical characteristics associated with a difference at a *p*-value < 0.20 were eligible for inclusion in the model. The adjusted odds ratios (ORs) and 95% confidence interval (CI) for treatment with CAZ-AVI with each outcome were then calculated. Odds ratios of overall in-hospital mortality for CAZ-AVI versus colistin among subpopulations of interest were also calculated. All statistical analyses were performed using STATA 18 (StataCorp LP, College Station, TX, USA).

## Figures and Tables

**Table 1:** Demographic and clinical characteristics of study patients *.

Characteristic	CAZ-AVI *n* = 89	IV Colistin *n* = 114	*p* Value
Demographic			
Age in years ^a^	62 ± 18	58 ± 18	0.070
Male	56 (62.9)	64 (56.1)	0.329
Comorbidity			
Cerebrovascular disease	24 (27)	25 (21.9)	0.405
Chronic heart failure	21 (23.6)	21 (18.4)	0.366
Chronic obstructive pulmonary disease	2 (2.2)	9 (7.9)	0.078
Connective tissue disease	3 (3.4)	5 (4.4)	0.712
Dementia	7 (7.9)	4 (3.5)	0.174
Diabetes mellitus	58 (65.2)	65 (57)	0.238
Hemiplegia or paraplegia	8 (9)	12 (10.5)	0.715
History of myocardial infarction	12 (13.5)	18 (15.8)	0.646
Hypertension	57 (64)	59 (51.8)	0.079
Immunosuppressed ^†^	26 (29.2)	31 (27.2)	0.751
Liver disease	3 (3.4)	6 (5.3)	0.516
Moderate to severe chronic renal failure	25 (28.1)	30 (26.3)	0.778
Neurological disease	20 (22.5)	18 (15.8)	0.226
Peptic ulcer disease	1 (1.1)	1 (0.9)	0.860
Peripheral vascular disease	13 (14.6)	11 (9.6)	0.278
Charlson comorbidity index ^b^	6 (3–8)	4 (2–7)	<0.001
Baseline serum creatinine in µmol/L ^b^	98 (55–200)	75 (58–118)	0.205
Baseline creatinine clearance in mL/min ^b^	58 (22–109)	75 (43–114)	0.183
Indwelling invasive devices			
Central venous catheter	43 (48.3)	85 (74.6)	<0.001
Foley catheter	51 (57.3)	74 (64.9)	0.269
Mechanical ventilation	35 (39.3)	49 (43)	0.600
Severity of illness			
Intensive care unit at infection onset	50 (56.2)	73 (64)	0.256
Sepsis	25 (28.1)	40 (35.1)	0.289
Septic shock	23 (25.8)	30 (26.3)	0.939
APACHE II score ^b^	21 (15–26)	20 (14–29)	0.853
Site of infection			
HAP	28 (31.5)	34 (29.8)	0.802
VAP	18 (20.2)	35 (30.7)	0.092
Wound	17 (19.1)	15 (13.2)	0.249
UTI	11 (12.4)	12 (10.5)	0.863
Intraabdominal	9 (10.1)	11 (9.6)	0.912
CLABSI	1 (1.1)	2 (1.8)	0.712
Other ^#^	5 (5.6)	5 (4.4)	0.687
Presence of bacteremia	10 (11.2)	33 (28.9)	0.002
Polymicrobial infection	42 (47.2)	55 (48.2)	0.881
Infectious diseases consultation	86 (96.6)	113 (99.1)	0.205
Time to active antibiotic (hours) ^b^	85 (16–120)	72 (10–144)	0.977
Time to study drug (hours) ^b^	100 (30–155)	72 (24–156)	0.201
Combination therapy ^+^	17 (19.1)	98 (86)	<0.001
Combination with more than one agent	2 (2.2)	13 (11.4)	0.013
Type of combination therapy			
IV Aminoglycoside	5	9	-
Aztreonam	9	2	-
Carbapenem	0	65	-
Cephalosporin	0	12	-
Fluoroquinolone	4	10	-
Inhaled aminoglycoside	1	1	-
Piperacillin/tazobactam	0	12	-
Susceptible to at least one combination antibiotic ^±^	9 (52.9)	48 (49)	0.763
Duration of therapy (days) ^b^	9 (6–14)	13 (8–16)	<0.001
Overall duration of hospitalization (days) ^b^	54 (27–101)	53 (29–112)	0.459

Abbreviation: APACHE II: Acute Physiology and Chronic Health Evaluation; CAZ-AVI: ceftazidime-avibactam; CLABSI: central line-associated bloodstream infection; HAP: hospital-acquired pneumonia; IV: intravenous; UTI: urinary tract infection; VAP: ventilator-associated pneumonia. ^a^ Mean ± standard deviation. ^b^ Median (interquartile range), otherwise, data are presented as *n* (%). * The χ^2^ test was used to compare categorical variables, whereas the independent *t*-test or Wilcoxon rank-sum test was used to compare continuous variables. ^†^ Neutropenic, chronic treatment with corticosteroids, active chemotherapeutic management of malignancy, or solid organ/bone marrow transplant patients on immunosuppressant therapy. ^#^ Including eight bacteremia of unknown origin, one meningitis, and one cystic fibrosis. ^+^ Given concurrently with the study drug for at least 48 h. ^±^ Among all isolates for patients who received combination therapy only.

**Table 2:** Outcomes in patients receiving ceftazidime-avibactam versus intravenous colistin.

Outcome ^a^	CAZ-AVI *n* = 89	IV Colistin *n* = 114	*p* Value	Odds Ratio (95% CI)	Adjusted Odds * Ratio (95% CI)
In-hospital mortality	32 (40)	56 (49.1)	0.060	0.58 (0.33–1.03)	0.61 (0.23–1.62)
Clinical cure	60 (67.4)	57 (50)	0.013	2.07 (1.16–3.68)	4.59 (1.65–12.74)
Acute kidney injury	13 (14.6)	49 (43)	<0.001	0.23 (0.11–0.45)	0.11 (0.04–0.31)
Risk	7	16	-	-	-
Injury	1	14	-	-	-
Failure	4	15	-	-	-
RRT	1	4	-	-	-
Microbiologic outcome ^b^					
Eradication	39 (61.9)	45 (51.1)	0.190	1.55 (0.80–2.99)	-
Persistence	24 (38.1)	43 (48.9)	-	-	-
Infection-related mortality	14 (15.7)	28 (24.6)	0.123	0.57 (0.28–1.17)	0.44 (0.15–1.28)
30-day readmission ^c^	11 (19.3)	6 (10.3)	0.176	-	-
30-day readmission due to infection ^c^	4 (7)	1 (1.7)	0.164	-	-
30-day recurrence ^c^	7 (12.3)	4 (6.9)	0.326	-	-
90-day recurrence ^c^	11 (19.3)	7 (12.1)	0.286	-	-
Length of hospital stay from onset of infection (days)	30 (16–52)	30 (20–68)	0.3177	-	-
Length of ICU stay from onset of infection (days) ^d^	21 (13–33)	22 (12–32)	0.822	-	-
Overall duration of mechanical ventilation (days) ^e^	22 (13–59)	22 (16–34)	0.894	-	-

Abbreviation: CAZ-AVI: ceftazidime-avibactam; CI: confidence interval; ICU: intensive care unit; RRT: renal replacement therapy. ^a^ Data are presented as *n* (%) or median (IQR); the χ^2^ test or Fisher exact test were used to compare categorical variables, whereas the independent *t*-test or Wilcoxon rank-sum test was used to compare continuous variables. ^b^ Only included patients who had repeated cultures (*n* = 63 in ceftazidime-avibactam arm and 88 in colistin arm). ^c^ Only included patients who survived (*n* = 57 in ceftazidime-avibactam arm and 58 in colistin arm). ^d^ Included only patients who were in the ICU at infection onset. ^e^ Only included patients who were mechanically ventilated during the infection episode. * Adjusted for age, the presence of bacteremia, combination therapy, central venous catheter, Charlson comorbidity index, ventilator-associated pneumonia, duration of therapy, baseline creatinine clearance, hypertension, chronic obstructive pulmonary disease, and dementia.

## Data Availability

The raw data supporting the conclusions of this article will be made available by the authors on request.
